# Case of Congenital Absence of the Globes of Both Eyes

**Published:** 1848-04

**Authors:** A. B. Williman


### RECORD OF MEDICAL SCIENCE.

*Case of Congenital Absence of the Globes of both Eyes.* By A. B. Williman, M. D.—The subject labouring under the above singular deformity, was presented to my notice, a few months past, by her owner Dr. Mazyck, at his plantation on the Santee river. She is about nine years of age, the last child of a black woman, who has reared a remarkably fine and healthy family, and presents in this relation, an exception to that law of hereditary transmission which establishes resemblance between parent and offspring, and most evidently observed in the features of the countenance. At birth, the infant was seen by Dr. Mazyck, and in a few days after by Dr. James Moultrie of this city, and to both of these gentlemen I am indebted for the following important information in relation to the case at that early period. Nothing unnatural was observed in the appearance of the face, except the firm adhesion of both eye-lids on their respective sides, when the attempt was made for the first time to open them. As the child appeared to suffer slight pain, no violence was used, and at the end of a week or ten days, there occurred a spontaneous separation for a few lines distance along the tarsal margins, very gentle force exercised with the fingers, soon completed the rupture of the remaining adherent portion, and the result disclosed the absence of the globes above-mentioned. Ever since the period here alluded to, (about two weeks after birth) the eye-lids have remained separated, and we may state from further recent examination, that these appendages, which are perfect in their formation and furnished with puncta and eye-lashes, are lined as usual with a healthy conjunctiva; this membrane extending throughout the entire inner surface of the orbit, was reflected rather firmly over a more resistant tissue deeply situated, (perhaps the rudiments of a sclerotic coat,) although beneath this, no protrusion was visible, and a probe passed over its surface could detect no openings. The existence of the orbicular muscles is evident, as their contraction rather inverts than elevates the lids, from the want of that point or fulcrum which is afforded by the globes in their normal state. It seemed also clear, after an examination of several days, that in both cavities there existed the secretion of a fluid resembling the tears, the lachrymal gland being in its normal position, and easily felt by slight pressure.

The orbitar margins are well developed below, but at their superior external portion appear somewhat deficient, giving to the superciliary ridges a slightly depressed form, and to the whole forehead a contracted character, often observed in persons deficient in intelligence. The girl, however, with the loss of the most important of the organs of sense, manifests a singular degree of intelligence in many others. Her disposition is cheerful; hearing is quite acute; and she possesses a remarkable power of distinguishing bodies by the touch, although not to that extent, as has been pretended, of forming ideas of colour in this manner.

As we shall probably forever be deprived of the means of verifying by post-mortem examination, (the girl living at too great a distance in the country,) to what extent the arrest of development may exist in the case just detailed, it now seems necessary to advert to some objections which may be entertained against the opinion of the present instance being one of original malformation.

The rarity of similar examples seems so great, as to demand a careful consideration of our case, before pronouncing on this point. In the extensive work of St. Geoffrey St. Hilaire, "Histoire des Anomalies de l'organization chez l'Homme et les Animaux," it is surprising, and not less matter of regret, that there should be no mention made of instances of malformation of the eyes, from an arrest of development; all his examples being a plurality of these organs in one orbit, or their singular position on other parts of the body. Such an account may also be found in Weller's "Maladies des Yeux," where he says:

"The seat of the eyes was not always the same ; sometimes they were found in their natural place, and again on remote situations, as, for instance, on the occiput, on the shoulders, the chest ; Shenk relates even having found them on the thighs." The only examples agreeing nearly with our own, have been related by the author cited above, (Weller) and are as follows : "Cases have been seen in which new born children possessed but one eye, and others, (which are the most frequent,) where they were completely deprived of them, with or without absence of the orbit. Amongst modern authors, Adam Schmidt relates the instance of a child deprived of eyes which lived six weeks. In making the autopsy, he found only the orbit, the lachrymal gland, the third pair of encephalic nerves, the first branch of the fifth pair, and the ophthalmic artery : the optic nerve was entirely wanting, and the foramen through which it ordinarily passes, was completely obliterated, or rather did not exist. We read in the same volume of this journal the history of a similar case, observed by Malacarne. The child which was the subject of it lived two months ; there was found in the orbit only the lachrymal gland and caruncula, the eye-lids and lachrymal canals."

Sufficient proof is here given, that children wanting the eyes from their arrest of development, have been instanced as living for some time after birth, and although we might infer so considerable defect in the bony structure of the forehead and anterior portions of the brain, as in common language to constitute a monstrosity, these are evidently not always requisite. Our case may be an example, together with those cited from Schmidt and Malacarne, where the orbit, lachrymal gland, and eye-lids were developed, with nothing unusual in the surrounding parts.

There is nothing to induce the suspicion in this case, that during fœtal life the eyes may have been destroyed by disease, such as syphilis or scrofula ; for in addition to such being a very rare occurrence, perhaps one unknown, the child exhibits not the least trace of these affections in its constitution, and this is equally true of the parents. The condition of all the other children being, as has been stated, remarkably healthy, is an additional objection to such a belief.

A complete absence of all inflammation, (and still more the violent form exhibited in the disease of purulent opthalmia) must dismiss the idea that this malady might have been the destroying cause of the eyes in the above case. It will be seen on reference to the details, that the tarsal margins were simply adherent, at the time of birth, that after the lapse of a few days when their separation occurred, no evacuation of pus nor the humours of the eye had taken place; when inspection was made of the parts hitherto concealed by the lids, the sclerotic coat showed the perfectly normal character, which may be seen at the present time, except that the position is at the most posterior part of the orbit, and gives no trace of a cornea, iris, nor any thing of the following description by Lawrence. "When the entire cornea has sloughed, and the humours of the eye have been evacuated, the tunics collapse, and the globe shrinks to one-third its original size, appearing as an opaque flattened tubercle."—*Charleston Med. Journ. and Review.*

